# Carotenoids in Marine Invertebrates Living along the Kuroshio Current Coast

**DOI:** 10.3390/md9081419

**Published:** 2011-08-22

**Authors:** Takashi Maoka, Naoshige Akimoto, Miyuki Tsushima, Sadao Komemushi, Takuma Mezaki, Fumihito Iwase, Yoshimitsu Takahashi, Naomi Sameshima, Miho Mori, Yoshikazu Sakagami

**Affiliations:** 1 Research Institute for Production Development, 15 Shimogamo-morimoto-cho, Sakyo-ku, Kyoto 606-0805, Japan; E-Mail: maoka@mbox.kyoto-inet.or.jp; 2 Graduate School of Pharmaceutical Sciences, Kyoto University, Yoshida-shimoadachi-cho, Sakyo-ku, Kyoto 606-8501, Japan; E-Mail: nmakimoto@leto.eonet.ne.jp; 3 Kyoto Pharmaceutical University, Misasagi Yamashina-Ku, Kyoto 607-8412, Japan; E-Mail: tsushima@mb.kyoto-phu.ac.jp; 4 Osaka City Graduate School of Engineering and Faculty of Engineering, Osaka City University, 3-3-138 Sugimoto, Sumiyoshi-ku, Osaka 558-8585, Japan; E-Mail: volvo.s80.clsssic386@gmail.com; 5 Kuroshio Biological Research Foundation, Nishidomari-560, Ootsuki-cho, Kochi 788-0333, Japan; E-Mails: mezaki@kuroshio.or.jp (T.M.); iwase@kuroshio.or.jp (F.I.); 6 Faculty of Agriculture, Kinki University, Nakamachi 3327-204, Nara-shi 631-8505, Nara, Japan; E-Mails: srdkt24134@hera.eonet.ne.jp (Y.T.); ato10min@yahoo.co.jp (N.S.); mori@nara.kindai.ac.jp (M.M.)

**Keywords:** carotenoid, marine invertebrates, food chain, metabolism

## Abstract

Carotenoids of the corals *Acropora japonica*, *A. secale*, and *A. hyacinthus*, the tridacnid clam *Tridacna squamosa*, the crown-of-thorns starfish *Acanthaster planci*, and the small sea snail *Drupella fragum* were investigated. The corals and the tridacnid clam are filter feeders and are associated with symbiotic zooxanthellae. Peridinin and pyrrhoxanthin, which originated from symbiotic zooxanthellae, were found to be major carotenoids in corals and the tridacnid clam. The crown-of-thorns starfish and the sea snail *D. fragum* are carnivorous and mainly feed on corals. Peridinin-3-acyl esters were major carotenoids in the sea snail *D. fragum*. On the other hand, ketocarotenoids such as 7,8-didehydroastaxanthin and astaxanthin were major carotenoids in the crown-of-thorns starfish. Carotenoids found in these marine animals closely reflected not only their metabolism but also their food chains.

## 1. Introduction

Marine animals, especially marine invertebrates, contain various carotenoids, with structural diversity [1–4]. Interesting structural carotenoids are still being found in marine animals [4]. In general, animals do not synthesize carotenoids *de novo*, and so those found in animals are either directly accumulated from food or partly modified through metabolic reactions [2]. The major metabolic conversions of carotenoids found in marine animals are oxidation, reduction, transformation of double bonds, oxidative cleavage of double bonds, and cleavage of epoxy bonds [2,3]. Therefore, various structural varieties are found in carotenoids of marine animals [4].

We have studied carotenoids in several marine invertebrates from chemical and comparative biochemical points of view [4]. In the present study, we focused on carotenoids of the corals *Acropora japonica*, *A. secale*, and *A. hyacinthus*, the tridacnid clam (elongate giant clam) *Tridacna squamosa*, crown-of-thorns starfish *Acanthaster planci*, and small sea snail *Drupella fragum*, inhabiting the Kuroshio current coast. These animals are closely associated within the food chain. Corals and the tridacnid clam are filter feeders and are associated with symbiotic zooxanthellae (dinoflagellate algae). On the other hand, the crown-of-thorns starfish and small sea snail *D. fragum* are carnivorous and mainly prey upon corals. Therefore, carotenoids that originated from zooxanthellae are passed to starfish and small sea snails through this food chain. In the present paper, we describe the carotenoids of these marine invertebrates.

## 2. Results and Discussion

**Figure 1.**
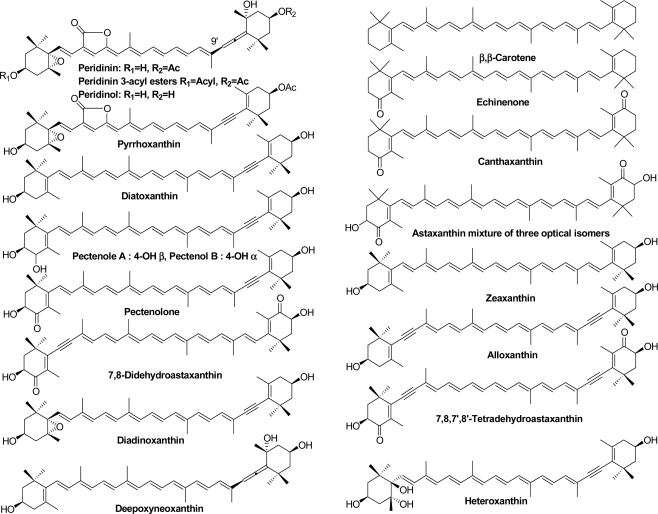
Carotenoids identified from *Acropora* corals, the tridacnid clam *T. squamosa*, starfish *A. planci*, and sea snail *D. fragum*.

Structural formulae of carotenoids identified from *Acropora* corals, the tridacnid clam *T. squamosa*, starfish *A. planci*, and sea snail *D. fragum* are shown in Figure 1.

### 2.1. Carotenoids of Corals and the Tridacnid Clam

The carotenoids composition of the corals and the tridacnid clam were similar to each other (Table 1). β,β-Carotene, peridinin (including the 9′*Z* isomer), pyrrhoxanthin, diatoxanthin, and diadinoxanthin were found in these animals. These carotenoid patterns resembled those of symbiotic zooxanthellae [5,6]. The results indicate that corals and the tridacnid clam directly absorb carotenoids from symbiotic zooxanthellae and accumulate them without metabolic modification. In the eggs of corals, peridinin and pyrroxanthin were present as major carotenoids. It was assumed that peridinin and pyrroxanthin play important roles in reproduction in corals, as with astaxanthin in salmonid fishes [7].

Recently, Daigo *et al.* studied carotenoids of more than 20 species of coral inhabiting reefs in Okinawa [8]. They reported that carotenoids found in these corals were not only peridinin and diadinoxanthin, that originated from symbiotic zooxanthellae, but also zeaxanthin, lutein, and, fucoxanthin, that originated from cyanobacteria, green algae, and diatoms. Cyanobacteria, green algae, and diatoms were epizoic and/or endolithic algae that grew in association with the corals. Corals accumulated carotenoids from these epizoic and/or endolithic algae [8]. However, the present study found that carotenoids in *Acropora* corals, inhabiting the Kuroshio current coast of Kochi, only consisted of those that originated from zooxanthellae. These differences might reflect the constitution of associating algae with corals.

Peridinin and pyrrhoxanthin were found to be major carotenoids in the tridacnid clam. In general, major carotenoids found in clams are fucoxanthin and its metabolites originating from diatoms [9–11]. On the other hand, neither fucoxanthin nor its metabolites were found in the tridacnid clam. This indicates that the tridacnid clam only ingested carotenoids from dinoflagellate algae. Similar results were reported in carotenoids of the bivalves, *Modiolus modiolus* and *Pecten maximus* [12].

### 2.2. Carotenoids of the Crown-of-Thorns Starfish

**Figure 2.**
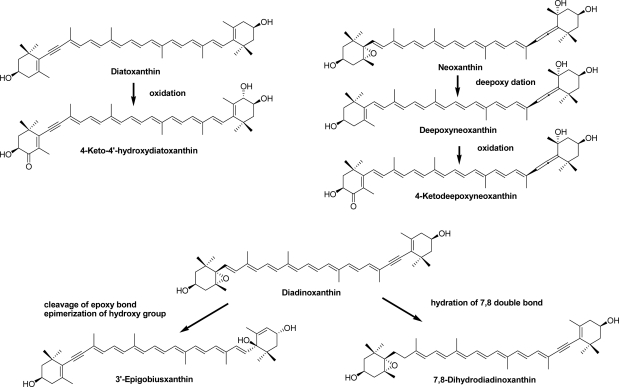
Possible bioformation roots of new carotenoids in crown-of-thorns starfish.

The crown-of-thorns starfish, *A. planci*, is a large, nocturnal sea star that mainly preys upon coral polyps. Like other starfish [13], 7,8-didehydroastaxanthin and astaxanthin were found to be major carotenoids along with pectenolone, 7,8,7′,8′-tetradehydroastaxanthin, diatoxanthin, and alloxanthin (Table 2). In general, the starfish can introduce a hydroxy group at C-3 and carbonyl group at C-4 in the β-end group of carotenoids [6]. So, 7,8-didehydroastaxanthin and astaxanthin were oxidative metabolites of diatoxanthin and β-carotene, respectively, ingested from dietary corals. Echinenone and canthaxanthin were metabolic intermediates from β,β-carotene to astaxanthin. The acetylenic carotenoids, pectenolone, pectenol A, and pectenol B, were also metabolic intermediates from diatoxanthin to 7,8-didehydroastaxanthin. Peridinol, one of the major carotenoids in the crown-of-thorns starfish, was converted from peridinin, which originated from corals, by hydrolysis. Furthermore, four new carotenoids; 4-ketodeepoxyneoxanthin, 4-keto-4′-hydroxydiatoxanthin, 3′-epigobiusxanthin, and 7,8-dihydrodiadinoxanthin, were isolated [14]. Details of the structural elucidation of those compounds were described previously [14]. In the present paper, the biosynthetic origins of these compounds are discussed (Figure 2). 4-Keto-4′-hydroxydiatoxanthin was one of the metabolic intermediates from diatoxanthin to 7,8-didehydroastaxanthin. 4-Ketodeepoxyneoxanthin might be an oxidative metabolite of deepoxyneoxanthin derived from neoxanthin by deepoxydation. 3′-Epigobiusxanthin might be derived from diadinoxanthin. 7,8-Dihydrodiadinoxanthin, which has a unique single bond in the 7,8-saturated polyene chain, may be a reduction metabolite of diadinoxanthin. Therefore, it was concluded that carotenoids ingested from corals were oxidatively metabolized and accumulated in the crown-of-thorns starfish.

### 2.3. Carotenoids of the Sea Snail *D. fragum*

Like the crown-of-thorns starfish, the small sea snail *D. fragum* also feeds on corals. The carotenoid composition of this snail resembled that of the dietary corals (Table 3). This indicated that *D. fragum* also accumulated carotenoids from dietary corals without metabolic modification, except for the esterification of peridinin. In the present study, peridinin 3-acyl esters were fully characterized based on ^1^H-NMR and FAB MS spectral data. The ^1^H-NMR signal of H-3 (δ 4.95), which showed 1.04 ppm downfield shift relative to the corresponding signal in peridinin [15,16], indicated that the hydroxy group at C-3 was acylated. Fatty acids esterified with peridinin were assigned as palmitic acid, palmitoleic acid, and myristic acid based on FAB-MS data. Previously, peridinol fatty acid ester was characterized by Moaka *et al*. [10] and Sugawara *et al*. [17]. However, peridinin 3-acyl esters have not yet been reported. The origin of zeaxanthin in this snail was unclear. It might have originated from associated algae such as cyanobacteria [8].

## 3. Experimental Section

### 3.1. General

The UV-Visible (UV-VIS) spectra were recorded with a Hitachi U-2001 in diethyl ether (Et_2_O). The positive ion FAB-MS spectra were recorded using a JEOL JMS-700 110A mass spectrometer with *m*-nitrobenzyl alcohol as a matrix. The ^1^H-NMR (500 MHz) spectra were measured with a Varian UNITY INOVA 500 spectrometer in CDCl_3_ with TMS as an internal standard. HPLC was performed on a Shimadzu LC-6AD with a Shimadzu SPD-6AV spectrophotometer set at 470 nm. The column used was a 250 × 10 mm i.d., 10 μm Cosmosil 5C18-II (Nacalai Tesque, Kyoto, Japan) with acetone:hexane (3:7, v/v) at a flow rate of 1.0 mL/min. The optical purity of astaxanthin was analyzed by chiral HPLC using a 300 × 8 mm i.d., 5 μm Sumichiral OA-2000 (Sumitomo Chemicals, Osaka, Japan) with *n*-hexane/CHCl_3_-ethanol (48:16:0.8, v/v) at a flow rate of 1.0 mL/min [18].

### 3.2. Animal Material

The corals *A. japonica*, *A. secale*, and *A. hyacinthus*, the tridacnid clam *T. squamosa*, the crown-of-thorns starfish *A. planc*, and the sea snail *D. fragum* were collected along the Ootsuki coast, Kochi Prefecture, Japan from July to August 2009 and 2010.

### 3.3. Analysis of Carotenoids

The extraction and identification of carotenoids were carried out according to our routine methods [19]. Carotenoids were extracted from living or fresh animal specimens with acetone. The acetone extract was transferred to ether-hexane (1:1) layer after the addition of water. The total carotenoid contents were calculated employing an extinction coefficient of $E_{\text{cm}}^{1\%}$ = 2100 (astaxanthin) [20] for the starfish *A. planci* and $E_{\text{cm}}^{1\%}$ = 1350 (peridinin) [20] for *A. japonica*, *T. squamosa*, and *D. fragum* at λ max. The ether-hexane solution was evaporated. The residue was subjected to HPLC on silica gel. Carotenoid compositions were estimated by the peak area of the HPLC on silica gel with acetone-hexane (3:7) monitored at 470 nm.

Individual carotenoids were identified by UV-VIS (ether), FAB MS, and partial ^1^H NMR (500 MHz, CDCl_3_).

### 3.4. Identification of Carotenoids

Identification of individual carotenoids were carried out on UV-VIS and FAB MS spectral data and compared with chromatographic property with authentic samples [19]. Optical isomer of astaxanthin in the crown-of-thorns starfish *Acanthaster planci* was analyzed by Chiral HPLC [18]. Astaxanthin fraction in *Acanthaster planci* was consisted of three optical isomers (3*R*,3′*R*):(3*R*,3′*S*):(3*S*,3′*S*) with a ratio of 32:14:54. Furthermore, peridiniol, peridinin and 9′*Z*-Peridinin were characterized by ^1^H NMR [15,16]. Structures of 7,8-ihydrodiadinoxanthin, 3′-epigobiusxanthin, 4-keto-4′-hydroxydiatoxanthin, 4-ketodeepoxyneoxanthin, and deepoxyneoxanthin were fully characterized by NMR [14].

### 3.5. Caracterization of Peridinin-3-acyl Esters

Peridinin-3-acyl esters. FAB-MS: *m/z* 868.5860 [M]^+^ (calcd. for C_55_H_80_O_8_, 868.5856) peridinin 3-palmitate, *m/z* 866.5698 [M]^+^ (calcd. for C_55_H_79_O_8_, 866.5703) peridinin 3-palmitolate, *m/z* 840.5550 [M]^+^ (calcd. for C_53_H_76_O_8_, 840.5547) peridinin 3-myristate; UV-VIS 455, 475 nm; ^1^H NMR (CDCl_3_), δ 0.88 (3H, t, *J* = 7.5 Hz, CH_3_ in fatty acid moiety), 0.99 (3H, s, H-16), 1.07 (3H, s, H-17′), 1.20 (3H, s H-17), 1.21 (3H, s H-18), 1.25 (about 24H, s, -CH_2_- in fatty acid moiety), 1.35 (3H, s, H-18′), 1.39 (3H, s, H-16′), 1.41 (1H, dd, *J* = 13, 7 Hz,H-2′β), 1.51 (1H, dd, *J* = 13, 12.5 Hz, H-4′β), 1.64 (1H, dd, *J* = 12.5, 12 Hz, H-2α eq), 1.79 (1H, dd, *J* = 12, 7 Hz, H-4β ax), 1.81 (3H, s, H-19′), 2.00 (H, ddd, *J* = 13, 4, 2 Hz, H-2′α), 2.04 (3H, s, CH_3_CO-), 2.23 (3H, s, H-20), 2.29 (1H, overlapped, H-α), 2.28 (2H, t, *J* = 7.5 Hz, -CH_2_-COOH in fatty acid moiety), 2.41 (1H, ddd, *J* = 14, 5, 1.5 Hz, H-4α), 4.95 (1H, m, H-3), 5.37 (1H, m, H-3′), 5.74 (1H, s, H-12), 6.06 (1H, s, H-8′), 6.11 (1H, d, *J* = 11 Hz, H-10′), 6.38 (1H, dg, *J* = 14, 11 Hz, H-11′), 6.40 (1H, d, *J* = 16 Hz, H-8), 6.46 (1H, d, *J* = 11 Hz, H-14′), 6.51 (1H, dd, *J* = 14, 11 Hz, H-15′), 6.61 (2H, dd, *J* = 14, 11 Hz, H-11′, 15′).

## 4. Conclusions

**Figure 3.**
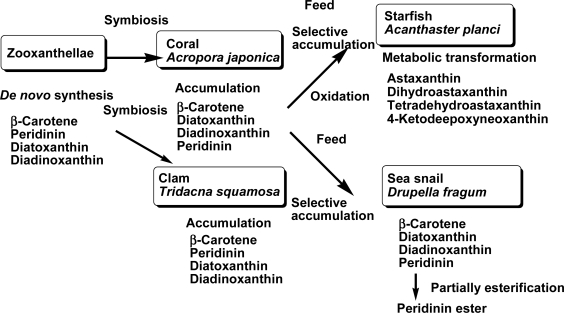
Accumulation and metabolism of carotenoids that originate from zooxanthellae to the starfish and sea snail through the food chain.

In conclusion, carotenoids found in the coral *A. japonica*, clam *T. squamosa*, starfish *A. planci*, and sea snail *D. fragum* well reflected not only their metabolism but also the food chain. The accumulation and metabolism of carotenoids that originate from zooxanthellae to the starfish through the food chain are summarized in Figure 3.

## Figures and Tables

**Table 1.** Carotenoids of *Acropora* corals and the tridacnid clam *Tridacna squamosa*.

	***Acropora japonica***		***A. secale***	***A. hyacinthus***	***Tridacna squamosa***

	**Whole**	**Egg**	**Whole**	**Whole**	**Mantle and foot**

Carotenoid content	3.3 (mg/100 g)	0.02	2.4	2.9	10

	composition (%)	%	%	%	%
β,β-Carotene	15.5	5.0	12.0	13.4	5.1
Diatoxanthin	5.5	15.0	4.5	5.2	0.9
Diadinoxanthin	4.5	5.0	5.0	5.5	9.2
Pyrrhoxanthin	45.5	20.0	50.6	40.5	10.1
Peridinin	13.0	50.0	10.0	16.0	44.1
9′Z-Peridinin	16.0	5.0	17.9	19.4	30.6

**Table 2.** Carotenoids of the crown-of-thorns starfish *Acanthaster planci.*

	**Whole**	**Gonad**

	0.46 mg/100 g	6.6 mg/100 g
β,β-Carotene	2.1	1.4
Echinenone	1.3	1.3
Canthaxanthin	1.6	1.6
7,8,7′,8′-Tetradehydroastaxanthin	2.0	1.6
7,8-Didehydroastaxanthin	35.6	35.3
Astaxanthin	9.8	5.8
Pectenolone	3.2	3.0
Diatoxanthin	3.2	15.8
Alloxanthin	3.2	11.8
Diadinoxanthin	3.0	8.6
7,8-Dihydrodiadinoxanthin	4.0	1.0
3′-Epigobiusxanthin	2.0	1.0
Pectenol A	2.0	1.6
Pectenol B	4.0	3.2
4-Keto-4′-hydroxydiatoxanthin	5.5	1.3
4-Ketodeepoxyneoxanthin	1.8	1.8
Deepoxyneoxanthin	1.0	0.3
Heteroxanthin	1.2	0.6
Peridinol	13.5	3.0

**Table 3.** Carotenoids of the sea snail *Drupella fragum*

**Carotenoid content**	**4.03 mg/100 g**

	composition (%)
β,β-Carotene	10.0
Peridinin-3-acyl esters	25.0
Zeaxanthin	15.0
Diatoxanthin	18.3
Diadinoxanthin	9.2
Pyrrhoxanthin	5.8
Peridinin	16.7
